# Dental Instruments

**Published:** 1902-12-15

**Authors:** J. A. Watling


### DENTAL INSTRUMENTS.

BY J. A. WATLING, D.D.S.

Discussion at Meeting of Southwestern Michigan Dental Society.

This matter of instruments is one to which I have given a good deal of attention for a number of years. Students go to college to learn how to use instruments, because as dentists they will be called upon to use them every day in their practice. It is therefore necessary that the student should learn to use them properly, for, unless they do so, they are not likely to learn after getting into practice. There is a great tendency at the present time for all operators, and especially the younger ones, to depend too much upon the engine in the preparation of cavities, some do to the exclusion of the hand excavator. This is a mistake, patients are always very grateful when the tooth is excavated without the use of the engine. The excessive use of the engine is generally brought about by the lack of good excavators.

At the present time they are made so imperfectly that they are almost worthless for excavating teeth. I am sure some members of the profession know little or nothing of what a sharp, cutting excavator is. This leads them to depend entirely upon the engine. I very frequently excavate a tooth from the start to the finish, especially in the incisors if the cavities are large, by the use of hand excavators only, and my patients are very grateful to me for doing so. For many years I made my own excavators, because I could make them better than the instrument makers at that time, and I find that even now if I want a really good instrument for a special purpose I can better make it myself than to buy it.

I want to call your attention to the hatchet form of excavator, which I use a great deal in anterior teeth. This instrument as made and put on the market is constructed on the cold-chisel plan instead of the knife-blade plan. The form of the cutting edge should be that of a knife blade and a little broader at the cutting edge, gradually narrowing to the neck. The hatchet and the hoe are the two upon which I largely depend for excavating the anterior teeth. For large mesial or distal cavities in the incisors I place the excavator just as far back in the healthy tooth substance as possible, and with one outward circular motion I take out just as much decay at possible. I repeat this for both the labial and lingual walls, and with two or three cuts I take out the greater portion of decay. The sensitive part of the tooth is just at the periphery of the dentine and if we can cut this out quickly we are doing a good service to the patient by having the pain so short in duration. This can all be done by the use of two forms of these excavators. Then by the use ot a smaller hoe sufficient undercuts can be made in the walls of the cavity for retention of the filling, slight undercuts without retaining pits as needed. It was formerly considered proper to make starting pits, but they are objectionable because they are made in the sensitive part of the tooth, and sometimes dangerously near the pulp. A tooth filled upon such preparation will remain sensitive for years. The enamel margins of the cavity should be beveled, the sharp edges should be trimmed so that the filling when completed in cavity will have something the appearance of a screw head.

In opening up crown cavities of upper molar teeth, instead of using a burr to enlarge the cavity by cutting away the hard enamel, which is a most unpleasant operation to the patient, take a small round gouge and set slightly back from the edge of the border of the cavity and with a slight rotary motion break down the overhanging enamel and enlarge the cavity to the desired extent. The operation is much quicker done than by an engine burr and it is also less expensive as it costs little to make such a chisel and it will outwear many burrs. This instrument may also be used successfully in approximal cavities of molars and bicuspids to break down the enamel or make dovetails. Instead of using a burr for preparing enamel margins I use four different-sized flat chisel-shaped instruments. Take this chisel between the thumb and finger and cut down the enamel border until it is of required strength. The instrument should be broad enough so there is no danger of slipping off and running into the gum. These same instruments also work very nicely at the cervical margin.

Another instrument which I value highly, is a large spoon-shaped excavator. I have quite frequently had men say to me, "Where do you use that? It is too large to do anything with." This is one of the most useful instruments in my office, it may be used in removing decay from very large cavities, for removing tartar and also for scraping down cement fillings. It is an all around useful instrument, at first sight many will think it too large to be of any use whatever.

Dr. E. A. Honey: The use of excavators, their forms, shapes and above all the manner of keeping them in good condition are very important questions. I frequently look over my instruments three or four times when I am in a hurry, to find one sharp enough to do the work necessary, and I think all of you have had the same experience and realize the importance of having them always in good condition. If any of you are ever in Kalamazoo and have the opportunity to visit Dr. Bannister I am sure you will not regret the time spent, he will teach you more in fifteen or twenty minutes about how to keep chisel and excavators sharp and in good condition, than you ever learned before. He forms and makes all his own chisels and excavators and has made a number for me and I must say they are the best I have ever been able to get anywhere. He has a little invention of his own for sharpening excavators, it is made so as to hold the excavator firmly in place, enabling one to get the blade sharpened at any required definite angle.

Dr. Rix: I want to emphasize the idea of every dentist knowing how to make his own instruments. Dentists of forty-five and fifty years ago had to make their own instruments, because it was very seldom that you could find one of proper form, they were not made in such numbers as to-day either, they were always very crude and not of good material. I have used instruments almost identical with those which Dr. Watling has exhibited, all my professional life, and I use them much more than I do the engine burrs. Wherever it is possible I do so, especially in large cavities, in molars or approximal cavities in the incisors, my method is much the same as that of Dr. Watling.

Dr. Honey: The invention of Dr. Bannister consists of a small metal block with set screws and clamps on its sides and an oil pad on its bottom. It is to slide over a stone hone which is about six inches long.

You have all experienced what a difficult matter it is to get a good edge on an excavator by means of the common hone, it is very apt to be at a wrong angle, and you endeavor to correct it and make it worse than ever. This appliance will hold your instrument at a definite angle at every point on the hone and a few sweeps will give you a nice clean blade. You can carry it so far on this hone as to remove the wire edge. The angle can be changed in a moment to accomodate different forms of the excavator. Dr. Bannister also makes drills, and he makes such drills as are far ahead of anything on the market to-day.

